# Rectal Plasmablastic Lymphoma in HIV/AIDS: Two Cases

**DOI:** 10.4021/wjon627w

**Published:** 2013-03-06

**Authors:** Alvaro Lopez-Iniguez, Mara Anais Llamas-Covarrubias, Guillermo Navarro-Blackaller, Ezequiel Velez-Gomez, Luz Alicia Gonzalez-Hernandez, Ariel Eduardo Campos-Loza, Fernando Amador-Lara, Jaime Federico Andrade-Villanueva

**Affiliations:** a HIV Department, Hospital Civil de Guadalajara Fray Antonio Alcalde, Guadalajara, Jalisco, Mexico; b Patology Department, Hospital Civil de Guadalajara Dr. Juan I. Menchaca. Guadalajara, Jalisco, Mexico

**Keywords:** AIDS, HAART, Plasmablastic lymphoma, Rectal tumor

## Abstract

Plasmablastic lymphoma is an aggressive variant of large B-cells lymphoma in which the infection by Human Immunodeficiency Virus and Epstein-Barr herpesvirus are involved. This recently denominated neoplasia has a special tropism through the oral cavity. However, its presence has been reported in the digestive tract, abdominal cavity and retroperitoneum. We describe two Human Immunodeficiency Virus infected patient cases with rectal presentation of PL in the HIV service of the Hospital Civil de Guadalajara.

## Introduction

Lymphoma is the second most frequent neoplasia on HIV infected patients and the most common variant is the Non-Hodgkin’s lymphoma. Plasmablastic lymphoma (PL) is a novel entity with an aggressive course; it predominantly occurs in the oral cavity and is associated with low response to the treatment. Furthermore, as opposite to the rest of lymphomas, PL develops in patients with CD-4 lymphocyte counts above 50 cells/µL. In recent years, a change in the course of PL has been observed, with an increasing number of non-oral occurrence, and the affection of immunocompetent subjects [1-3].

## Case Report

### Case 1

A 37-year-old male diagnosed with HIV-1 infection 2 months before, coursing in C2 AIDS stage (CD4+ lymphocytes with 242 cells, 7%), under treatment with Efavirenz, Tenofovir/Emtricitabine and anti-tuberculosis therapy due to history of lymphatic tuberculosis. In spite of treatment, the patient continued losing weight, which markedly increased during the last month. Two weeks prior hospitalization he presented jaundice, asthenia, adynamia, nausea, vomit and dehydration; all these being the cause of consult. During the physical exploration severe dehydration, generalized jaundice and hepatalgia were found, as well as an anal lump that extended to the rectum and was overlooked by the patient despite being a mass of approximately 5 × 10 cm. The patient denied rectal symptoms. This case was approached as a suspicion of toxic hepatitis probably secondary to anti-TB treatment and as a rectal mass in study. The hepatotoxic medication was removed and toxic hepatitis symptoms disappeared.

**Figure 1:**
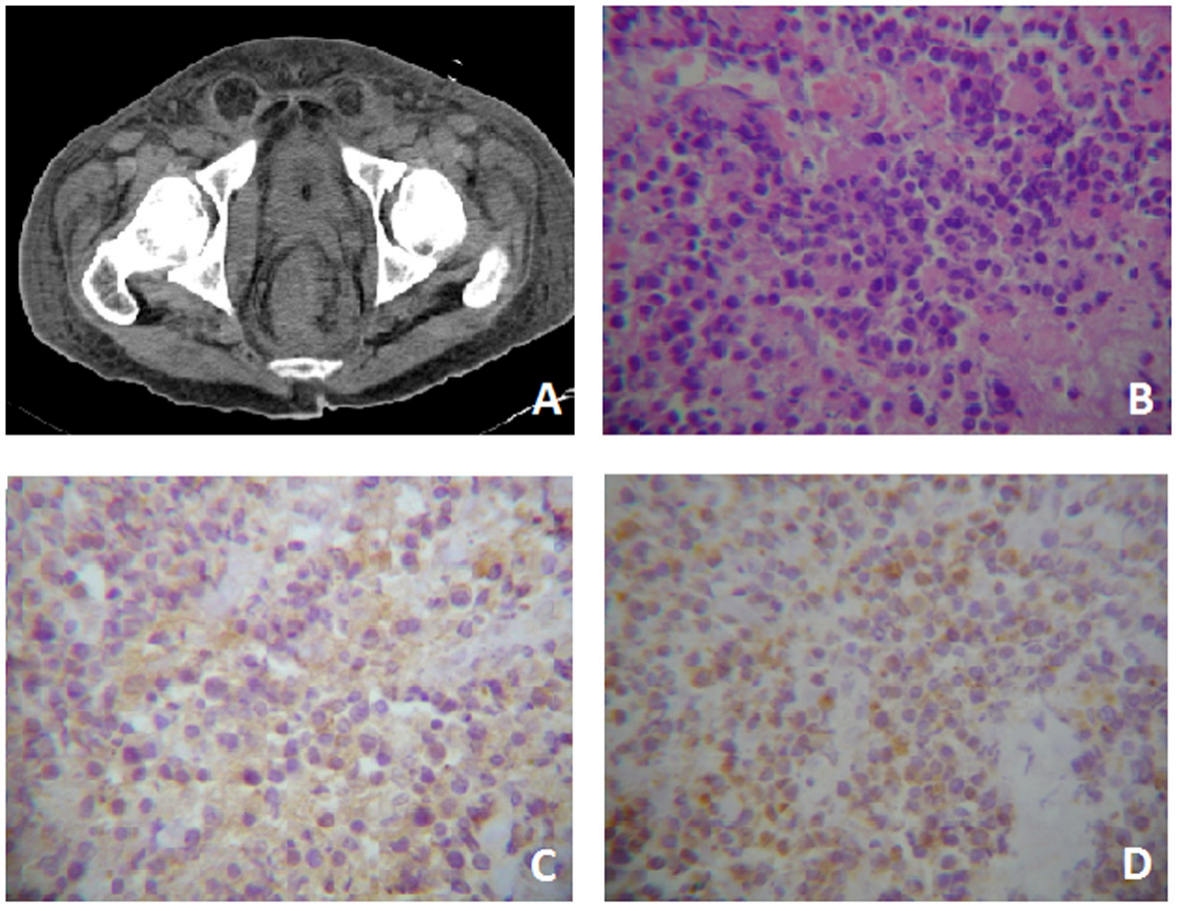
Case One. A) Pelvis CT where evidences rectal tumor with perirectal fascia engrossed. B) Tumor with extensive necrosis in the right lower quadrant, viable zones of monotonous, slightly cohesive, moderately pleomorphic plamacytoids showing a paranuclear light halo. HE (400 ×). C) Immunreactivity for CD-20 cytoplasmatic and membranous (400 ×). D) Immunohistochemistry for CD-138 membranous and cytoplasmatic (400 ×).

On the other hand the rectal exam showed an irregular and not actively bleeding mass in the rectal blister. Computerized Axial Tomography (CT) was performed, and the presence of a mass comprising the rectum and anus, with infiltration to the perirectal fascia and distortion of the architecture with extension to the bladder was demonstrated (Fig. 1A). Laboratory tests showed abnormal levels of lactate dehydrogenase (LDH) (561 UI/L; reference: 91 - 190 UI/L) and β-2 microglobulin (11.8 g/dl; reference: 0 - 3 g/dl).

An ulcerated, necrotic and friable tumor was evidenced by colonoscopy, and a sample of six small fragments (approximately 2 mm diameter each) was obtained for histopathology studies. No other lesion was detected in the rest of the colon.

Histopathological studies reported highly necrotizing neoplasia that showed a monotonous large cell pattern of lymph-shaped, eccentric nuclei, prominent nucleoli and abundant pale cytoplasm cells in its deepest portion. Immunohistochemistry showed membrane and cytoplamatic positivity to CD-20 and CD-138 and a nuclear Ki-60 on 60% of the cells (Fig. 1B-D). Bone marrow aspiration showed no evidence of tumor infiltration. A high presence of gamma serum paraprotein (37.1 g/dl; reference: 12 - 22 g/dl) was reported. The patient was treated with EPOCH-R. Nonetheless, during his hospital stay he developed a urinal tract infection that subsequently complicated as urosepsis. Urine culture revealed the presence of multi-drug resistant *Pseudomonas aeruginosa* and despite antimicrobial treatment the patient died.

### Case 2

A 31-year-old patient diagnosed with infection by HIV-1 AIDS stage C3 (CD4+ lymphocytes count with 93 cells, 4.5%) 3 months before. At the time of presentation he was being treated with lopinavir/ritonavir and tenofovir/emtricitabine. The reason of consult is the presence of a 2 × 2 × 1 cm mass in the perianal region. Medical exploration revealed an anal condylomata and an indurated, irregular and painful tumor without active bleeding. Nevertheless, cytology studies reported an unspecific inflammatory process negative to malignancy. Since the patient was asymptomatic and according to cytology results, expectant management was established.

During the 5 subsequent months, the patient experienced difficulty defecating and urinating, as well as the appearance of a non-painful mass in the hypogastrium. Nonetheless, the patient waited a month before seeking for medical attention, due to symptomatology exacerbation and severe rectorrhagia which lead to hypovolemic shock and an acute renal lesion grade III. The patient was hospitalized and an abdominal, indurated and painless mass from the pubic symphysis to the umbilical scar was detected. During the abdomino-pelvic CT an abdominal tumor with recto-vesical component was found, compressing the moving ureters and causing bilateral hydronephrosis; adding a postrenal damage to the etiology of the acute renal lesion. Due to the severity of the clinical picture, a nephrostomy catheter was placed in the right kidney, and therapy of renal substitution and hemodialisis were then initiated.

**Figure 2:**
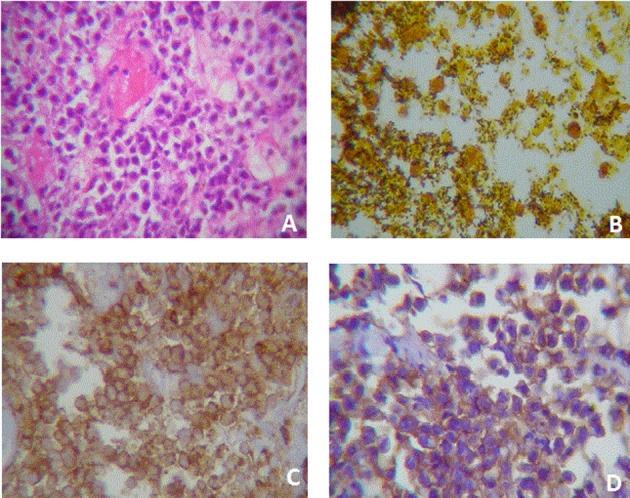
Case two. A) Plasmacytoid cells with eccentric nucleus and a light paranuclear zone. Cells are slightly cohesive and some nucleus are wheel-shaped. It is also evident the presence of a fibrin microthrombi. HE (400X). B) Abundant bacillus colonization. Bacteria are black stained. Whartin Starry (100X). C) Membrane immunoreactivity to CD-45 (400X). D) Membrane an cytoplasmic immunoreactivity to CD-138.

Biopsy was performed and 10 samples (diameter approx. 2 mm) were taken. Microscopic observation showed intense necrosis and abundant bacillus colonization at the surface, as well as monotonous eccentric nuclei, prominent nucleoli and abundant pale cytoplasmatic tumor cells at the bottom; consistent with PL. Bone marrow aspiration was also performed and no malignant infiltration was evidenced (Fig. 2). Chemotherapy was initiated with the CHOP scheme and the patient received 4 cycles. As a result, the tumoral mass was reduced, the acute lesion solved, and regular function was recovered, so hemodialisis was removed.

Clinical improvement was sustained for 3 months, after that, the patient relapsed showing urinating difficulty and fetid rectorrhea. Three more masses were detected: one in the armpit, another one in the thorax and the last one in the left iliac crest; all of them smaller than 2 cm, indurated, non-painful. Growth of a hypogastric tumor involving the descending colon and small intestine was confirmed by CT. According to the clinical picture, radiotherapy was initiated, however, no improvement was obtained and the patient finally died.

## Discussion

PL is a rare malignancy predominantly described on patients with HIV/AIDS. Its incidence is unknown but some series have reported it as the 2.6% of all lymphomas. It is a disease with a recent description, classified as an aggressive subtype of diffuse B-cells lymphoma with an immunophenotype of plasmatic cells. PL is not associated with race or sex and it mainly affects any race or people between the 3rd and 5th decade of life. Of interest, unlike the rest of HIV related lymphomas, PL may present in patients with CD-4 lymphocytes counts above of 50 cells/µL.

The majority of case descriptions have been in the oral cavity, although it also affects the digestive tract, lymph nodes, retroperitoneum and skin. The reason for oral cavity tropism is unknown. HAART has been defined as a modifying factor of PL’s natural course, as its use is associated with an increase of extra oral PL presentation. [1, 2, 4-10].

There is a strong association of PL to EBV infection which can be evidenced by serological tests and in-situ hybridization for the EBER particle; nevertheless, we were not able to perform these tests on the cases described. On the other hand, LMP1 immunohistochemistry in case 2 turned out negative, probably due to the low sensitivity of this study.

Microscopic observation of biopsies showed oval, abundant cytoplasm and prominent nucleoli cells, derived from mutated B-lymphocytes in a reactive lymphocytes cell background, showing a pattern of starry sky. In both presented cases, the cells showed plasmacytoid characteristics as defined by eccentric nucleus and car wheel disposition of chromatin, giving a plasmablastic aspect. Characteristically, PL expresses plasmatic cell markers being VS38c, CD-38, CD-138 and oncogene type 1 of the multiple myeloma (MUM-1) the most common, although the expression of CD20, CD79 and the PAX5 protein has also been associated [2, 3].

In this report, the demonstration of CD-45 is relevant since it is useful for distinguish PL from the rest of plasmatic cells neoplasias. One of our cases was reactive for CD-20 consistent to the B cell origin of the tumor. So far there are 150 PL cases reported on literature, from which rectal presentation has been the less with only one case reported [11]. We have presented 2 rectal lumps cases with extensive necrosis on AIDS patients.

PL prognosis is obscure since life expectancy has not been reported beyond 14 months even with intensive chemotherapeutic treatment. By means of CHOP, EPOCH or Hyper-CVAD schemes, combined with radiotherapy only minimal effects have been obtained. Treatment with Rituximab is not indicated, except for those CD-20 positive tumors, however it does not affect survival. On HIV-1 infected patients, antiretroviral treatment has demonstrated an improvement on survival although not significative [10, 12].
